# Dynamics of SARS-CoV-2 Transmission Among Indian Nationals Evacuated From Iran

**DOI:** 10.1017/dmp.2020.393

**Published:** 2020-10-22

**Authors:** Suman Saurabh, Ritesh Kumar, Nitesh Kumar, Pankaj Bhardwaj, Vijaya Lakshmi Nag, Mahendra Kumar Garg, Sanjeev Misra

**Affiliations:** Department of Community Medicine and Family Medicine, All India Institute of Medical Sciences (AIIMS), Jodhpur, Rajasthan, India; Engineering Science Laboratory – Central Scientific Instruments Organization (CSIO), Council for Scientific and Industrial Research (CSIR), Chandigarh, India; Department of Microbiology, All India Institute of Medical Sciences (AIIMS), Jodhpur, Rajasthan, India; Department of General Medicine, All India Institute of Medical Sciences (AIIMS), Jodhpur, Rajasthan, India; Director, All India Institute of Medical Sciences (AIIMS), Jodhpur, Rajasthan, India

**Keywords:** COVID-19, evacuation, quarantine, *R*0, SARS-CoV-2

## Abstract

**Objective:**

During the coronavirus disease (COVID-19) pandemic, Indian nationals evacuated from Iran were quarantined at Jaisalmer, Rajasthan. We wished to study the transmission of severe acute respiratory syndrome coronavirus 2 (SARS-CoV-2) in this closed population.

**Methods:**

A basic susceptible, exposed, infected, and removed (SEIR) compartmental model was developed using the daily stepwise approach in Microsoft Excel. An advanced model using standard differential equations in Python software version 3.6 was used to estimate *R*_0_ based on model fit to actual data.

**Results:**

Forty-eight SARS-CoV-2 infections were found among the 474 quarantined individuals. Out of these, 44 (92%) were asymptomatic. *R*_0_ for the overall duration was found to be 2.29 (95% CI: 1.84–2.78). Male gender and age ≥ 60 years were associated with SARS-CoV-2 infection (RR = 4.33, 95% CI: 2.07–9.05 and 5.32, 95% CI: 3.13–9.04, respectively). Isolation of infected individuals and stricter quarantine of remaining individuals reduced the *R*_0_ from 2.41 initially to 1.17 subsequently.

**Conclusion:**

*R*_0_ value was found comparable to the earlier studies indicating similar transmission dynamics among quarantined individuals in India. Universal testing and prompt isolation of infected individuals reduced the transmission of SARS-CoV-2. Smaller group sizes should be preferred to large groups during facility-based quarantine in evacuation situations. The role of asymptomatic individuals appears to be strong in SARS-CoV-2 transmission within closed populations.

## INTRODUCTION

As of November 17, 2020, the coronavirus disease (COVID-19) pandemic caused by SARS-CoV-2 has resulted in around 53.7 million cases globally and 1.3 million deaths.^1^ India is now a major epicenter of the pandemic with 8.8 million cumulative cases, exceeded only by the United States. It is imperative to understand the epidemiology and transmission dynamics of the infection, in order to guide the prevention strategy. Outbreaks in closed susceptible populations such as the *Diamond Princess* cruise ship have provided an opportunity to understand the transmission of SARS-CoV-2.^2-7^

India had restricted international travel in March 2020 to several COVID-19 affected countries, including Iran. Subsequently, it was decided to evacuate the Indian nationals residing abroad who were affected as a result of these travel restrictions. The evacuated individuals were universally screened for SARS-CoV-2 infection by rRT-PCR testing prior to departure, and symptomatic screening was done upon their arrival in India on March 14–15, 2020. Subsequently, they were quarantined at the facility at Jaisalmer, Rajasthan.

Upon completion of 14 days of quarantine, all individuals irrespective of symptoms were again tested for SARS-CoV-2 infection by rRT-PCR, from March 28–April 1, 2020. Repeat testing of those who had tested negative initially was conducted after April 1, 2020. Those who tested positive were admitted in isolation wards at All India Institute of Medical Sciences (AIIMS), Jodhpur, Rajasthan. They were further tested twice on days 14 and 15 of initial positive test results as per extant national guidelines applicable prior to May 8, 2020.^8^ They were discharged and allowed to travel to their home districts in India if both the test results were found negative. We studied the transmission dynamics of SARS-CoV-2 infection in this closed population.

## METHODS

We used the compartmental SEIR model, wherein S, E, I, R respectively denote the susceptible, the exposed, the infected, and removed components of the closed population.^2^ To begin with, all individuals were considered susceptible. Those testing positive at the quarantine facility were considered to move from “susceptible” to “infected” compartment on the day of sample collection and stayed there till the day of being isolated, which was usually a day after the declaration of the test result. From the date of isolation and onward, they were considered “removed.” The total number N = S + E + I + R remained fixed at each step. The input parameters of the model were first specified in time steps of 1 day starting from March 14, 2020. It was subsequently applied to a stepwise prediction model in Microsoft Excel and standard differential equation model in Python software version 3.6.^2^ The differential equations specifying the instantaneous rates of change with respect to time (t) in the S, E, I, and R compartments were as follows:
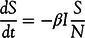


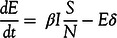


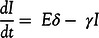


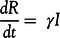



Here, δ refers to the reciprocal of the incubation period equivalent to the rate of exposed turning infectious per day. Similarly, *γ* refers to the rate of infectious individuals recovering per day (1/D). The relationship between *R*_0_ (basic reproduction number) and duration of infectiousness (D) is expressed as per the following equation:
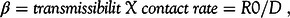
where β referred to the transmissibility multiplied by the contact rate or number of secondary cases generated by an infectious case per day.

### Stepwise Model in Microsoft Excel

The objective of this basic model was to use backward extrapolation in the stepwise compartmental model to provide an estimate of initially infected individuals while considering known infection transmission parameters.

A mean *R*_0_ value of 3.28 was taken from the mean of values reported by previous studies.^9^ A duration of infectiousness of 10 days yielded the β as 0.328.^10^ The median incubation period was taken as 5.5 days.^10^ Thus, δ and *γ* values were taken as 0.182/day and 0.1/day, respectively, for the basic stepwise model. This initial number of infected individuals at day 1 was varied so as to achieve the observed number of SARS-CoV-2 positive individuals till all the individuals were tested by April 1, 2020 (day 19).

### Differential Equation Model in Python v3.6

The standard differential equations of the SEIR model were first specified in Python software v.3.6.^2^ The model was optimized to provide the best fit, using the maximum likelihood approach with the input parameters so that the value of *R*0 could be estimated. Further, in order to visualize the effect of removal of positives and stricter avoidance of mixing on transmission, the *R*0 value was assessed separately for the first wave when all individuals had been tested and subsequently when repeat testing was done. The prior values of δ, *γ*, and *R*0 were the same as in the basic model.^10^

## RESULTS

We reported the SARS-CoV-2 transmission among 474 individuals (53.6% males and 46.4% females) who were accommodated together in dormitories of the quarantine facility of Jaisalmer. All the evacuees were Muslim and more than half (268, 56.5%) belonged to Jammu and Kashmir followed by Maharashtra (13.1%), Rajasthan (9.7%), Uttar Pradesh (4.9%), Telangana (4.2%), Karnataka (2.5%), West Bengal (1.5%), Gujarat (1.3%), Jharkhand (1.3%), and other states (5.1%). Nearly half (47.9%) belonged to the 15–29 years age group and 21.7% were age 60 years or older (Table 1). Male gender and age > 60 years were significantly associated with being infected with SARS-CoV-2 (Table 2).

**TABLE 1:** Age and Gender Distribution of the Individuals Evacuated From Iran During the COVID-19 Pandemic and Quarantined at Jaisalmer, India

Age Category (in years)	Female		Male		Total	
	N	%	N	%	N	%
0–14	6	2.7	2	0.8	8	1.7
15–29	126	57.3	101	39.8	227	47.9
30–44	17	7.7	50	19.7	67	14.1
45–59	29	13.2	40	15.7	69	14.6
60 Plus	42	19.1	61	24.0	103	21.7
Grand Total	**220**	**100.0**	**254**	**100.0**	**474**	**100.0**

**TABLE 2:** Association of Age and Gender With SARS-CoV-2 Infection Among Individuals Evacuated From Iran During the COVID-19 Pandemic and Quarantined at Jaisalmer, India

Characteristics	SARS-CoV-2 Positive n/total (%)	SARS-CoV-2 Negative n/total (%)	Risk Ratio (95% CI)	*P*-value
**Gender**				
Male	40/254 (15.7)	214/254 (84.3)	4.33 (2.07–9.05)	< 0.0001
Female	8/220 (3.6)	212/220 (96.4)	–	
**Age Group**				
60 Years and more	28/103 (27.2)	75/103 (72.8)	5.32 (3.13–9.04)	< 0.0001
Up to 60 years	20/371 (5.4)	351/371 (94.6)	–	

**FIGURE 1:**
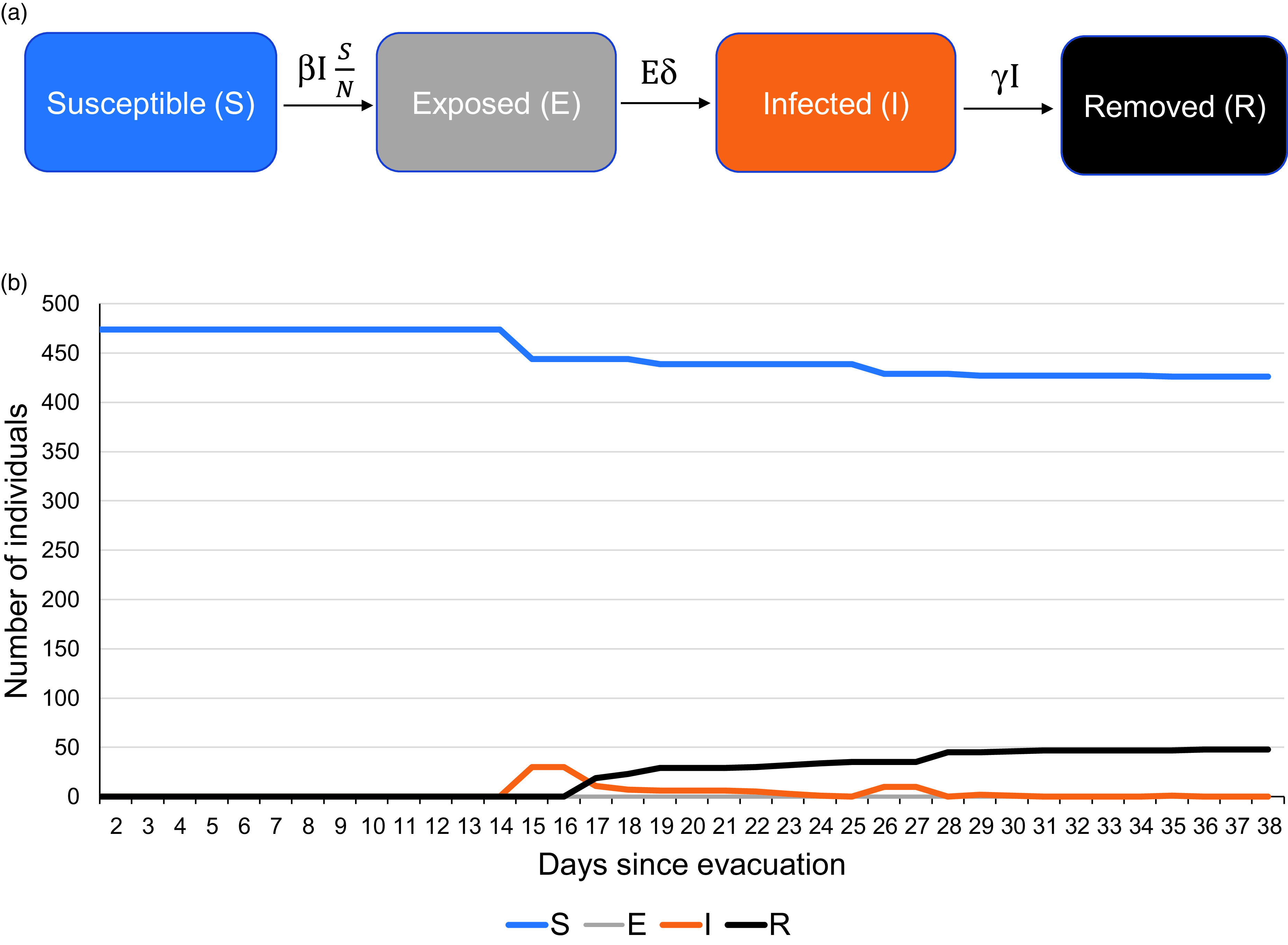
SEIR Compartmental Model for Individuals Evacuated and Quarantined at Jaisalmer, India, During the COVID-19 Pandemic: (a) Schematic Description and (b) Number of Individuals in Each Compartment Since the Evacuation.

In the first wave of testing from March 28, 2020–April 1, 2020, all 474 quarantined individuals were tested irrespective of symptoms. Thirty-five were detected as positive in the first wave (Figure 1). Subsequently, repeat testing among those who had tested negative earlier had detected 13 new infections (see Figure 1). Therefore, a total of 48 individuals were eventually found infected with SARS-CoV-2 (see Figure 1; Supplementary File 1). Only 4 (8.3%) among them were found to have a fever. No other symptoms were reported. The mean stay in hospital isolation for the infected individuals was 15.5 ± 3.6 days.

The stepwise model estimated that an initial size of 11 infected individuals (2.3% of total) was sufficient to result in 35 infected individuals by April 1, 2020, by the time the first wave of testing was completed (Supplementary File 2).

**FIGURE 2:**
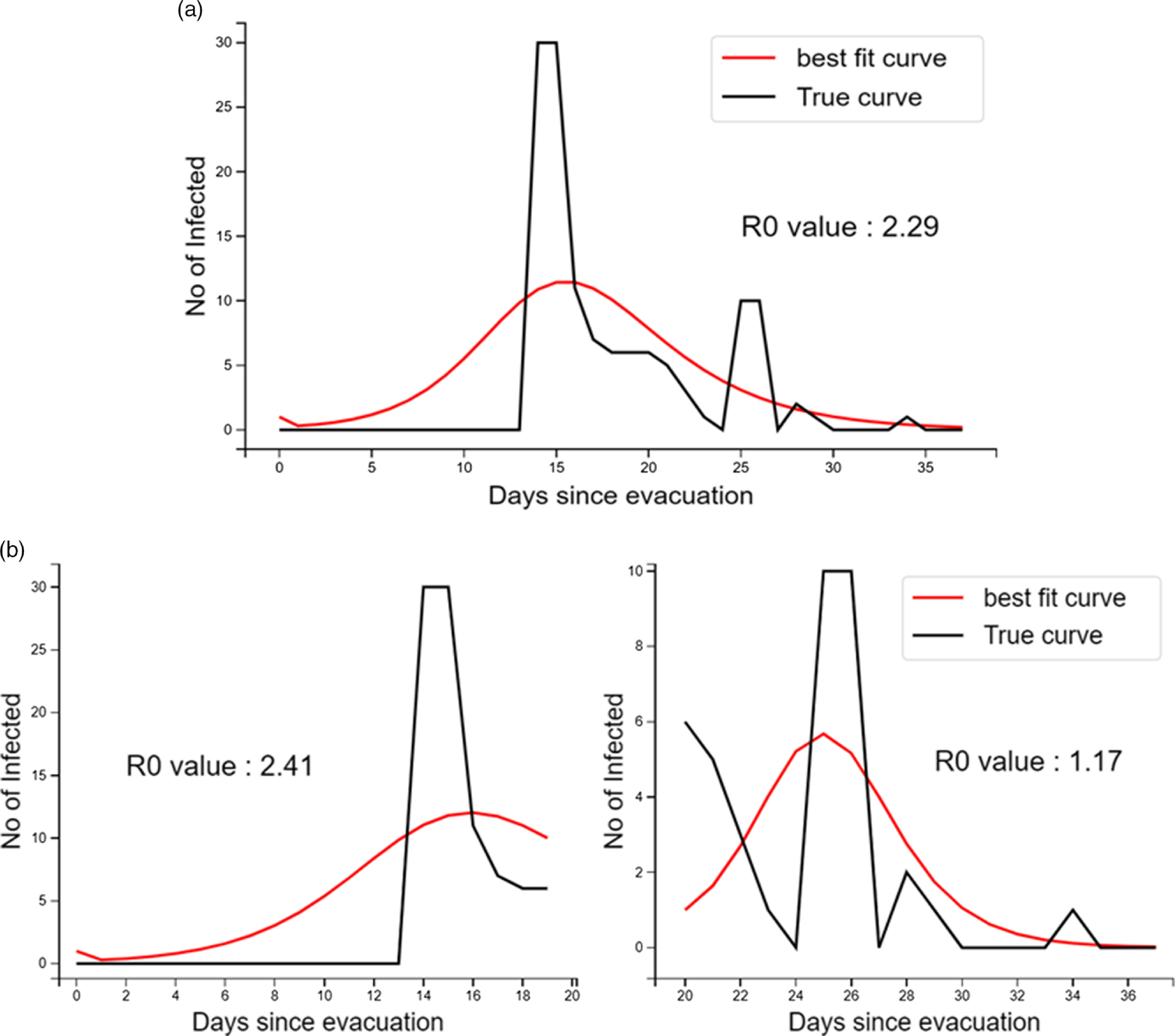
Number of Infected Individuals Evacuated and Quarantined at Jaisalmer, India, During the COVID-19 Pandemic – Observed and Fitted to Gamma Distribution and R_0_: (a) Throughout the Transmission Period and (b) Stratified During the First Wave of Testing and Later.

In the differential equation model, the overall *R*0 was estimated to be 2.29 (95% CI: 1.84–2.78). For the individuals detected positive in the first wave of testing, the *R*0 value was 2.41 (95% CI: 0.53–4.86), which subsequently reduced to 1.17 (95% CI: 0.87–1.24). The best fit curve of infected individuals overall and separately in the first and second waves was also obtained (Figure 2). Further details of the Python 3.6 code used for analysis are provided in Supplementary File 3.

## DISCUSSION

### Factors Influencing SARS-CoV-2 Transmission

Male gender and older age were found to be significantly associated with SARS-CoV-2 infection. Although male gender and older age have been found to be at risk of severe COVID-19,^11,12^ it remains unclear whether they are also at higher risk of SARS-CoV-2 infection. The gender and age difference in infection in a small group, such as in the present study, could mostly be attributed to assortative mixing of men of the older age group in the quarantine facility, rather than male gender and older age being true risk factors of SARS-CoV-2 infection. Further, sociocultural practices, such as the use of a cloth face covering of *hijab* or *burka* among Muslim women, could reduce their risk of infection by providing a partial barrier to touching and infective aerosol exposure of the eyes, nose, and face.^13^ This could be applicable in the present study as all the evacuated individuals were Muslim.

For larger populations, individual-level risk factors related to the biological aspect of age and gender may be more pertinent rather than behavioral, cultural, and specific contextual factors. A greater risk of viral infections among the elderly could be attributed to a disruption of both innate and adaptive immunity along with an increased production of inflammatory mediators.^14^ Gender differences in the COVID-19 infection and other infections have also been widely attributed to underlying differences in the genetic makeup of males and females.^15^

The overall *R*0 value of 2.29 found in our study was comparable to previous estimates reported mainly from China in the early phase of the COVID-19 pandemic.^9^ The reduction in the *R*0 estimate, once infected individuals were isolated and stricter physical separation was ensured, was similar to the finding from the *Diamond Princess* cruise ship.^2,16^ During the early phase of the pandemic, embarkation of passengers on ships at specific ports had enabled the ascertainment of the index case with reasonable accuracy.^5^ On the other hand, it becomes difficult to establish the index case for evacuations when all the individuals usually start traveling together from the origin country.

### Universal Screening Prior to Evacuation

Universal screening for SARS-CoV-2 infection was conducted prior to evacuation. However, the evacuation situation during outbreaks provides a unique challenge wherein susceptible individuals are brought within confined spaces during transit to the airport and to the quarantine facility. Therefore, the stage of the outbreak of the country from where evacuation is being carried out appears to be important. If evacuation is done during peak SARS-CoV-2 transmission, there would be a greater risk of infecting more susceptible individuals during the process. Consequently, stricter safeguards would be needed. Therefore, the decision to evacuate must carefully take into account these epidemiological factors.

Further, the rationale of universal screening prior to evacuation mainly depends on the test having perfect accuracy and having the ability to detect all individuals shedding the virus. The rRT-PCR test being used for screening has been estimated to have 2–29% false-negatives in different scenarios.^17^ The evacuation happened from Iran when the COVID-19 outbreak was well established and the prevalence of SARS-CoV-2 infection and concurrently the pre-test probability of positive rRT-PCR test could be considered to be high. For example, a realistic scenario of 5% pre-test positive probability during the outbreak^18^ could lead to 1.6% individuals testing false-negatives, assuming 70% sensitivity and 95% specificity of the rRT-PCR test.^19^ This was similar to the stepwise model estimation of 2.3% evacuated individuals having undetected SARS-CoV-2 infection upon arrival to India.

### Quarantine of Evacuated Individuals

Mathematical modeling of lockdown in a community has revealed that only very strict quarantine and smaller household sizes may succeed in curbing the transmission.^20^ Similarly, facility-based quarantine is likely to be effective only when individuals are accommodated separately in smaller groups of up to 5–6 individuals in rooms rather than in large groups in dormitories. Therefore, where feasible, the option of home quarantine should be used in order to achieve smaller group sizes.

Due to the considerable proportion of asymptomatic SARS-CoV-2 infections, mandatory screening of all individuals irrespective of symptoms upon completion of quarantine appeared to be the right decision. We found that another 13 quarantined individuals who were found negative in the first wave of testing turned positive on repeat testing. It is likely that they were incubating when the testing was done initially for all individuals. Therefore, once transmission has started, even testing all quarantined individuals at a single point of time might not prove sufficient. Hence, it becomes all the more important to maintain adequate physical separation, ensure sufficient hand hygiene, and ensure strict avoidance of mixing to prevent the initial flare-up of transmission during quarantine.

### Role of Asymptomatic Individuals

Our observation of around 90% asymptomatic SARS-CoV-2 infection matches the higher bound of 18–88% found in prior studies.^3,7,21,22^ It also further supports the role of asymptomatic or mildly symptomatic carriers in transmitting the SARS-CoV-2 infection.^23^ Asymptomatic transmission in COVID-19 has been implicated to lead to undetected transmission and is therefore responsible for large outbreaks.^24^ An increasing recognition of the role of asymptomatic transmission has since lead to stricter recommendations for universal mask-wearing in areas with suspected transmission, high population density, or where social distancing is not feasible.^25^

It is notable that India introduced provisions for home isolation of only asymptomatic and mildly symptomatic SARS-CoV-2 infected individuals on May 10, 2020.^26^ Prior to this, all infected individuals were supposed to be either admitted in hospital and isolated at facilities and were to be discharged only upon testing negative twice.^26^ An increasing recognition of the role of asymptomatic or pre-symptomatic individuals in the transmission of acute respiratory diseases can help the decision makers accept home quarantine and home isolation strategies much earlier in the course of future epidemics. Community engagement to involve next-door neighbors and frontline health workers should be adopted for monitoring and ensuring effective home quarantine and isolation. Facility-based quarantine and isolation should preferably be used in special situations related to foreign travel, especially in the early phase of the outbreak. This is since it would not be feasible to monitor home isolation in a limited number of individuals who would require ongoing domestic travel to reach their homes, widely dispersed throughout a large country such as India. Further, there would be risk of infection associated with domestic travel.

### Limitations

Existing modeling techniques are well suited to the input of daily incidence data, which are easy to obtain for symptomatic cases. Our study had the limitation that daily testing and incidence data could not be used in the situation of mass testing of individuals irrespective of symptoms. Also, our population size was smaller, and we had a lesser number of data points. Therefore, we had comparatively larger error estimates for the time-stratified *R*0 values. Further, *R0* calculations based on incidence data of infected individuals would be accurate only if the diagnostic test used had near 100% sensitivity and specificity, that is, no infections are missed. In a scenario of 70% sensitivity of rRT-PCR,^19^ the *R0* value would be underestimated as many infections might be missed even after conducting a universal screening. Therefore, we suggest that future studies on disease transmission invariably take accuracy of the diagnostic test in consideration. We also didn’t have detailed information on quarantine locations and age and gender-wise group sizes of quarantined individuals. This could have helped explain whether older males were more affected due to assortative mixing or due to their biological susceptibility.

## CONCLUSIONS

The *R*_0_ estimate for SARS-CoV-2 infection found in the present study indicates a similar transmission pattern as in China during the early phase of the pandemic in January 2020. In addition to universal screening prior to evacuation, smaller group sizes should be preferred during quarantine to help limit SARS-CoV-2 transmission. Further, after the completion of quarantine, all individuals irrespective of symptoms should again be tested and those found to be infected should be promptly isolated. The role of asymptomatic individuals in SARS-CoV-2 transmission appears to be strong in the context of large-scale quarantine measures. Therefore, universal screening in closed populations appear to be more effective as compared to symptomatic screening alone.

## ABBREVIATIONS

AIIMS: All India Institute of Medical Sciences
COVID-19: coronavirus disease
*R*0: basic reproduction number
rRT-PCR: real-time reverse transcription polymerase chain reaction
SARS-CoV-2: severe acute respiratory syndrome coronavirus 2
SEIR: susceptible, exposed, infected, and removed (*or* recovered)

## Supplementary material

For supplementary material accompanying this paper visit https://doi.org/10.1017/dmp.2020.393. click here to view supplementary material
