# Supplementary material for: Dynamics of SARS-CoV-2 Transmission Among Indian Nationals Evacuated From Iran
Source: Disaster Med Public Health Prep. 2020 Oct 22:1–7. doi: 10.1017/dmp.2020.393 (PMC7737123; doi:10.1017/dmp.2020.393)
Supplement: Supplementary file 1 [file S1935789320003936sup.zip › S1935789320003936sup002.html.htm]

Jaisalmer\_SEIR\_modelling


Make sure to open in Colab to see the plots!

In [31]:

```
from scipy.integrate import odeint
from scipy import optimize
import numpy as np
import matplotlib.pyplot as plt
import pandas as pd
%matplotlib inline 
#!pip install mpld3
#import mpld3
#mpld3.enable_notebook()
```

In [28]:

```
def plotseird(t, S, E, I, R, D=None, L=None, R0=None, Alpha=None, CFR=None):
  f, ax = plt.subplots(1,1,figsize=(10,4))
  ax.plot(t, S, 'b', alpha=0.7, linewidth=2, label='Susceptible')
  ax.plot(t, E, 'y', alpha=0.7, linewidth=2, label='Exposed')
  ax.plot(t, I, 'r', alpha=0.7, linewidth=2, label='Infected')
  ax.plot(t, R, 'g', alpha=0.7, linewidth=2, label='Recovered')
  if D is not None:
    ax.plot(t, D, 'k', alpha=0.7, linewidth=2, label='Dead')
    ax.plot(t, S+E+I+R+D, 'c--', alpha=0.7, linewidth=2, label='Total')
  else:
    ax.plot(t, S+E+I+R, 'c--', alpha=0.7, linewidth=2, label='Total')

  ax.set_xlabel('Time (days)')

  ax.yaxis.set_tick_params(length=0)
  ax.xaxis.set_tick_params(length=0)
  ax.grid(b=True, which='major', c='w', lw=2, ls='-')
  legend = ax.legend(borderpad=2.0)
  legend.get_frame().set_alpha(0.5)
  for spine in ('top', 'right', 'bottom', 'left'):
      ax.spines[spine].set_visible(False)
  if L is not None:
      plt.title("Lockdown after {} days".format(L))
  plt.show();

  if R0 is not None or CFR is not None:
    f = plt.figure(figsize=(12,4))
  
  if R0 is not None:
    # sp1
    ax1 = f.add_subplot(121)
    ax1.plot(t, R0, 'b--', alpha=0.7, linewidth=2, label='R_0')

    ax1.set_xlabel('Time (days)')
    ax1.title.set_text('R_0 over time')
    # ax.set_ylabel('Number (1000s)')
    # ax.set_ylim(0,1.2)
    ax1.yaxis.set_tick_params(length=0)
    ax1.xaxis.set_tick_params(length=0)
    ax1.grid(b=True, which='major', c='w', lw=2, ls='-')
    legend = ax1.legend()
    legend.get_frame().set_alpha(0.5)
    for spine in ('top', 'right', 'bottom', 'left'):
      ax.spines[spine].set_visible(False)

  if Alpha is not None:
    # sp2
    ax2 = f.add_subplot(122)
    ax2.plot(t, Alpha, 'r--', alpha=0.7, linewidth=2, label='alpha')

    ax2.set_xlabel('Time (days)')
    ax2.title.set_text('fatality rate over time')
    # ax.set_ylabel('Number (1000s)')
    # ax.set_ylim(0,1.2)
    ax2.yaxis.set_tick_params(length=0)
    ax2.xaxis.set_tick_params(length=0)
    ax2.grid(b=True, which='major', c='w', lw=2, ls='-')
    legend = ax2.legend()
    legend.get_frame().set_alpha(0.5)
    for spine in ('top', 'right', 'bottom', 'left'):
      ax.spines[spine].set_visible(False)

    plt.show();
```

## Programming the Exposed-Compartment¶

In [3]:

```
def deriv(y, t, N, beta, gamma, delta):
    S, E, I, R = y
    dSdt = -beta * S * I / N
    dEdt = beta * S * I / N - delta * E
    dIdt = delta * E - gamma * I
    dRdt = gamma * I
    return dSdt, dEdt, dIdt, dRdt
```

In [4]:

```
def deriv1(y, t, N, R0):
    S, E, I, R = y
    dSdt = -R0* 0.1 * S * I / N
    dEdt = R0*0.1 * S * I / N - (1/5.5) * E
    dIdt = (1/5.5) * E - 0.1 * I
    dRdt = 0.1 * I
    return dSdt, dEdt, dIdt, dRdt
```

In [5]:

```
def deriv2(y, t, N, R0, gamma, delta):
    S, E, I, R = y
    dSdt = -R0*gamma * S * I / N
    dEdt = R0*gamma * S * I / N - delta * E
    dIdt = delta * E - gamma * I
    dRdt = gamma * I
    return dSdt, dEdt, dIdt, dRdt
```

In [10]:

```
N = 474
D = 10.0 # infections lasts ten days
gamma = 1.0 / D # fix it 0.1
delta = 1.0 / 5.5 # incubation period of five.five days
R_0 = 3.28
beta = R_0 * gamma  # R_0 = beta / gamma, so beta = R_0 * gamma
S0, E0, I0, R0 = N-1, 0, 1, 0  # initial conditions: one exposed
```

In [7]:

```
t = np.linspace(0, 100, 100) # Grid of time points (in days)
y0 = S0, E0, I0, R0 # Initial conditions vector
```

In [11]:

```
# Integrate the SIR equations over the time grid, t.
ret = odeint(deriv, y0, t, args=(N, beta, gamma, delta))
S, E, I, R = ret.T
```

In [ ]:

```
# Integrate the SIR equations over the time grid, t.
ret1 = odeint(deriv1, y0, t, args  = (N, R0))
S1, E1, I1, R1 = ret1.T
```

In [8]:

```
# Integrate the SIR equations over the time grid, t.
ret2 = odeint(deriv2, y0, t, args  = (N,  R0, gamma, delta))
S2, E2, I2, R2 = ret2.T
```

In [32]:

```
plotseird(t, S, E, I, R)
```

In [ ]:

```
plotseird(t, S1, E1, I1, R1)
```

In [9]:

```
plotseird(t, S2, E2, I2, R2)#
```

In [13]:

```
# get the real data
# fit the curve
data = pd.read_csv('SEIR_Jaisalmer_new.csv')
print(data)
print(data.shape)
```

```
    Day        Date    S  E   I   R  N (total)  Daily incidence
0     1  14/03/2020  474  0   0   0        474                0
1     2  15/03/2020  474  0   0   0        474                0
2     3  16/03/2020  474  0   0   0        474                0
3     4  17/03/2020  474  0   0   0        474                0
4     5  18/03/2020  474  0   0   0        474                0
5     6  19/03/2020  474  0   0   0        474                0
6     7  20/03/2020  474  0   0   0        474                0
7     8  21/03/2020  474  0   0   0        474                0
8     9  22/03/2020  474  0   0   0        474                0
9    10  23/03/2020  474  0   0   0        474                0
10   11  24/03/2020  474  0   0   0        474                0
11   12  25/03/2020  474  0   0   0        474                0
12   13  26/03/2020  474  0   0   0        474                0
13   14  27/03/2020  474  0   0   0        474                0
14   15  28/03/2020  444  0  30   0        474               30
15   16  29/03/2020  444  0  30   0        474                0
16   17  30/03/2020  444  0  11  19        474                0
17   18  31/03/2020  444  0   7  23        474                0
18   19  01/04/2020  439  0   6  29        474                5
19   20  02/04/2020  439  0   6  29        474                0
20   21  03/04/2020  439  0   6  29        474                0
21   22  04/04/2020  439  0   5  30        474                0
22   23  05/04/2020  439  0   3  32        474                0
23   24  06/04/2020  439  0   1  34        474                0
24   25  07/04/2020  439  0   0  35        474                0
25   26  08/04/2020  429  0  10  35        474               10
26   27  09/04/2020  429  0  10  35        474                0
27   28  10/04/2020  429  0   0  45        474                0
28   29  11/04/2020  427  0   2  45        474                2
29   30  12/04/2020  427  0   1  46        474                0
30   31  13/04/2020  427  0   0  47        474                0
31   32  14/04/2020  427  0   0  47        474                0
32   33  15/04/2020  427  0   0  47        474                0
33   34  16/04/2020  427  0   0  47        474                0
34   35  17/04/2020  426  0   1  47        474                1
35   36  18/04/2020  426  0   0  48        474                0
36   37  19/04/2020  426  0   0  48        474                0
37   38  20/04/2020  426  0   0  48        474                0
(38, 8)
```

In [14]:

```
def fit_odeint(x, beta, gamma,delta):
    return odeint(deriv, y0, x, args=(N, beta, gamma, delta))[:,2]
```

In [15]:

```
def fit_odeint1(x, R0):
    return odeint(deriv2, y0, x, args=(N, R0))[:,2]
```

In [45]:

```
def optimize_sliced_data(x,y):
# Do the sampling 100 times to get the error estimates 
    popt, pcov = optimize.curve_fit(fit_odeint, x, y)
    fitted = fit_odeint(x, *popt)
    return popt, pcov, fitted
def show_parameters(popt, pcov):
    print(f'beta: {popt[0]}')
    print(f'gamma: {popt[1]}')
    print(f'delta: {popt[2]}')
    perr = np.sqrt(np.diag(pcov))
    print('Covariance Matrix', pcov)
    print('Error estimates', perr)
    #print(type(popt))
    R0 = popt[0]/popt[1]
    print("R0 value : %2.4f" %(R0) )
    return popt[0], popt[1],perr, pcov, R0 

def optimize_sliced_data1(x,y):
# Do the sampling 100 times to get the error estimates 
    popt, pcov = optimize.curve_fit(fit_odeint1, x, y)
    return popt, pcov
```

In [17]:

```
x = [i for i in range(0,data.shape[0])]
y = np.asarray(data['I'])
```

In [ ]:

```
x1  = x[0:20]
y1 = y[0:20]

popt11, pcov11 =  optimize_sliced_data1(x1,y1)
#beta1,gamma1, errors1,cov1, R01 = show_parameters(popt1, pcov1) 
#print(popt1[0]+1.95596*np.sqrt(pcov1[0,0]))
print(popt11)
```

In [18]:

```
x1  = x[0:20]
y1 = y[0:20]

popt1, pcov1, fit =  optimize_sliced_data(x1,y1)

beta1,gamma1, errors1,cov1, R01 = show_parameters(popt1, pcov1) 
print(popt1[0]+1.95596*np.sqrt(pcov1[0,0]))
```

```
beta: 6.07047306718135
gamma: 2.5204630441600466
delta: 0.3285719963902595
Covariance Matrix [[ 2556.10997174   186.41629999  -191.09982516]
 [  186.41629999    13.99529647   -13.83706332]
 [ -191.09982516   -13.83706332    14.31438618]]
Error estimates [ 50.55798623   3.7410288    3.78343576]
R0 value : 2.4085
104.959871816
```

```
C:\Users\rk19abb\AppData\Local\conda\conda\envs\test\lib\site-packages\scipy\integrate\odepack.py:218: ODEintWarning: Excess work done on this call (perhaps wrong Dfun type). Run with full_output = 1 to get quantitative information.
  warnings.warn(warning_msg, ODEintWarning)
```

In [34]:

```
x2  = x[20:]
y2 = y[20:]

popt2, pcov2 =  optimize_sliced_data(x2,y2)
beta2,gamma2, errors2,cov2, R02 = show_parameters(popt2, pcov2) 
#print('95% Confidence interval' popt2[0]-1.95596*np.sqrt(pcov2[0,0]))
```

```
beta: 4.351646508333826
gamma: 3.7265403400082553
delta: 66.22611649049372
Covariance Matrix [[  0.17833252   0.16427229  -3.12403477]
 [  0.16427229   0.16529856  -2.88603573]
 [ -3.12403477  -2.88603573  67.1730262 ]]
Error estimates [ 0.42229435  0.40656926  8.19591521]
R0 value : 1.1677
```

```
C:\Users\rk19abb\AppData\Local\conda\conda\envs\test\lib\site-packages\scipy\integrate\odepack.py:218: ODEintWarning: Excess work done on this call (perhaps wrong Dfun type). Run with full_output = 1 to get quantitative information.
  warnings.warn(warning_msg, ODEintWarning)
```

In [59]:

```
#print(fitted)
#plt.plot(x, y, '-')
#plt.plot(x, fitted, '-')
#plt.show()

# prepare confidence level curves
#nstd = 1.0 # to draw 5-sigma intervals
#popt_up = popt + nstd*perr
#popt_dw = popt – nstd*perr
#popt_dw= popt-nstd*perr
fit1 = fit_odeint(x1, *popt1)
fit2= fit_odeint(x2, *popt2)
#fit_up = fit_odeint(x, *popt_up)
#fit_dw = fit_odeint(x, *popt_dw)
#print(fit_dw)
#print(fit_up)
#plot
fig, (ax1,ax2) = plt.subplots(1,2, figsize = (12, 5))

ax1.set_xlabel('Days since evacuation', fontsize=18)
ax1.set_ylabel('No of Infected', fontsize=18)
#ax1.set_title('fit with only Infected' , fontsize=18)
ax1.plot(x1, fit1, 'r', lw=2, label='best fit curve')
ax1.plot(x1, y1, 'k-', lw=2, label='True curve')
#plt.plot(x,y,'k-', lw=2)
#ax.fill_between(x,fit_up, fit_dw, alpha = 0.25)
#ax.fill_between(x, fit_up, fit_dw, alpha=.25, label='5-sigma interval')

ax1.text(2, 15, 'R0 value : %2.2f' %(R01) , style='italic',fontsize=20)
       #bbox={'facecolor': 'red', 'alpha': 0.5, 'pad': 10})

ax2.set_xlabel('Days since evacuation', fontsize=18)
ax2.set_ylabel('No of Infected', fontsize=18)
#ax2.set_title('fit with only Infected' , fontsize=18)
ax2.plot(x2, fit2, 'r', lw=2, label='best fit curve')
ax2.plot(x2, y2, 'k-', lw=2, label='True curve')
#plt.plot(x,y,'k-', lw=2)
#ax.fill_between(x,fit_up, fit_dw, alpha = 0.25)
#ax.fill_between(x, fit_up, fit_dw, alpha=.25, label='5-sigma interval')

ax2.legend(loc='upper right',fontsize=18)
ax2.text(29, 4.7, 'R0 value : %2.2f' %(R02) , style='italic',fontsize = 20)
       #bbox={'facecolor': 'red', 'alpha': 0.5, 'pad': 10})
plt.tight_layout()
plt.savefig('Initial_and_final.png')
plt.show()
```

In [24]:

```
# take all the data
popt, pcov =  optimize_sliced_data(x,y)
beta, gamma, errors,cov, R0 = show_parameters(popt, pcov)
```

```
beta: 6.08561453568913
gamma: 2.6602082911733196
delta: 0.3641120878925249
Covariance Matrix [[ 113.68976598   14.03440916   -8.28702721]
 [  14.03440916    1.83410849   -1.00151256]
 [  -8.28702721   -1.00151256    0.60982964]]
Error estimates [ 10.66254032   1.35429262   0.7809159 ]
R0 value : 2.2876
95% confidence Interval -14.7698878198
```

In [63]:

```
fit = fit_odeint(x, *popt)
#fit_up = fit_odeint(x, *popt_up)
#fit_dw = fit_odeint(x, *popt_dw)
#print(fit_dw)
#print(fit_up)
#plot
fig, (ax1) = plt.subplots(1,1, figsize = (8, 5))

ax1.set_xlabel('Days since evacuation', fontsize=18)
ax1.set_ylabel('No of Infected', fontsize=18)
#ax1.set_title('fit with only Infected' , fontsize=18)
ax1.plot(x, fit, 'r', lw=2, label='best fit curve')
ax1.plot(x, y, 'k-', lw=2, label='True curve')
#plt.plot(x,y,'k-', lw=2)
#ax.fill_between(x,fit_up, fit_dw, alpha = 0.25)
#ax.fill_between(x, fit_up, fit_dw, alpha=.25, label='5-sigma interval')

ax1.text(25, 15, 'R0 value : %2.2f' %(R0) , style='italic', fontsize  = 20)
       #bbox={'facecolor': 'red', 'alpha': 0.5, 'pad': 10})


ax1.legend(loc='upper right',fontsize=18)
plt.tight_layout()
plt.savefig('Overall.png')
plt.show()
```
